# Impaired microvascular reactivity after eccentric muscle contractions is not restored by acute ingestion of antioxidants or dietary nitrate

**DOI:** 10.14814/phy2.14162

**Published:** 2019-07-10

**Authors:** Ryan G. Larsen, Jens M. Thomsen, Rogerio P. Hirata, Rudi Steffensen, Eva R. Poulsen, Jens B. Frøkjær, Thomas Graven‐Nielsen

**Affiliations:** ^1^ Sports Sciences, Department of Health Science and Technology Aalborg University Aalborg Denmark; ^2^ Department of Health Science and Technology SMI, Aalborg University Aalborg Denmark; ^3^ Department of Clinical Immunology Aalborg University Hospital Aalborg Denmark; ^4^ Department of Radiology Aalborg University Hospital Aalborg Denmark; ^5^ Department of Clinical Medicine Aalborg University Aalborg Denmark; ^6^ Center for Neuroplasticity and Pain (CNAP), Department of Health Science and Technology SMI, Aalborg University Aalborg Denmark

**Keywords:** Beetroot juice, blood oxygen level dependent (BOLD), hyperemia, muscle damage, nitric oxide, oxidative stress

## Abstract

Unaccustomed eccentric exercise leads to impaired microvascular function but the underlying mechanism is unknown. In this study, we evaluated the role of oxidative stress and of nitric oxide (NO) bioavailability. Thirty young men and women performed eccentric contractions of the tibialis anterior (TA) muscle (ECC), with the contralateral leg serving as nonexercising control (CON). Participants were randomized into three groups ingesting an antioxidant cocktail (AO), beetroot juice (BR) or placebo 46 h postexercise. At baseline and 48 h postexercise, hyperemic responses to brief muscle contractions and 5 min of cuff occlusion were assessed bilaterally in the TA muscles using blood oxygen level dependent (BOLD) magnetic resonance imaging. Eccentric contractions resulted in delayed time‐to‐peak (~22%; *P* < 0.001), blunted peak (~21%; *P* < 0.001) and prolonged time‐to‐half relaxation (~12%, *P* < 0.001) in the BOLD response to brief contractions, with no effects of AO or BR, and no changes in CON. Postocclusive time‐to‐peak was also delayed (~54%; *P* < 0.001) in ECC, with no effects of AO or BR, and no changes in CON. Impaired microvascular reactivity after eccentric contractions is confined to the exercised tissue, and is not restored with acute ingestion of AO or BR. Impairments in microvascular reactivity after unaccustomed eccentric contractions may result from structural changes within the microvasculature that can diminish muscle blood flow regulation during intermittent activities requiring prompt adjustments in oxygen delivery.

## Introduction

Muscle work involving unaccustomed eccentric contractions is known to result in pain, inflammation, and an overall decline in muscle function (Clarkson and Hubal, 2002). Although less investigated, there is evidence to suggest impaired vascular function following unaccustomed eccentric exercise (Barnes et al., 2010; Stacy et al., 2013; Caldwell et al., 2016). Results of impaired vascular function have also been extended to the microvasculature with reports of attenuated vasodilation in response to adenosine (Heap et al., (2006)) and slowed hemodynamics at the onset of muscle contractions (Kano et al., 2005). Using blood oxygen level‐dependent (BOLD) magnetic resonance (MR) imaging, we recently extended these results by showing a slowed contraction‐induced hyperemic response in the microvasculature 24 h and 48 h after unaccustomed eccentric contractions of human tibialis anterior (TA) muscle (Larsen et al., 2015). Notably, the hyperemic response was preserved in the contralateral, nonexercising TA muscle, suggesting a localized effect of eccentric contractions on microvascular reactivity.

The underlying mechanism for slowed reactivity of the microvasculature after eccentric exercise is not resolved. However, eccentric exercise is accompanied by inflammation and generation of reactive oxygen species (ROS) that may primarily derive from the microvasculature within the exercised muscle tissue (Hellsten et al., 1997). Accumulation of ROS can result in oxidative stress (Tidball, 2005; Barnes et al., 2010), defined as increased activity of ROS relative to the capacity of the antioxidant defense systems (Tidball, 2005; Sindler et al., 2014). Several lines of evidence support a link between elevated oxidative stress and reduced vascular function (Jablonski et al., 2007; Richardson et al., 2007; Donato et al., 2010; Limberg et al., 2013; Sindler et al., 2014). Specifically, oxidative stress may impair vasodilation by diminishing the synthesis and release of vasodilatory agents from the endothelium (Jablonski et al., 2007; Sindler et al., 2014). In particular, nitric oxide (NO) produced via NO synthase is scavenged by excess ROS which lowers NO bioavailability (Sindler et al., 2014). Among many functions, NO plays an important role in smooth muscle cell relaxation, and lower NO bioavailability is linked with reduced vascular endothelial function (Hobbs et al., 2013; Sindler et al., 2014).

Supporting the notion of a causal link between oxidative stress and vascular function, several studies have shown that antioxidants (AO) acutely improve vascular function (Eskurza et al., 2004; Jablonski et al., 2007; Crecelius et al., 2010; Donato et al., 2010; Limberg et al., 2013). Specifically, oral ingestion of an AO cocktail consisting of vitamin C, vitamin E and alpha‐lipoic acid has proven effective in reducing plasma free radicals (Richardson et al., 2007; Donato et al., 2010; Wray et al., 2012) and acutely restoring vascular function in individuals with elevated oxidative stress (Richardson et al., 2007; Donato et al., 2010; Wray et al., 2011; Wray et al., 2012). Hence, this AO cocktail may also be effective in scavenging free radicals and acutely restoring microvascular function after eccentric exercise. The nitrate–nitrite–NO pathway is an alternative source of NO, which supports NO signaling during conditions of impaired endogenous NO production (Lundberg et al., 2009). Ingestion of beetroot juice (BR) acutely elevates plasma levels of nitrate (NO_3_^‐^) and nitrite (Wylie et al., 2013), and presumably improves vascular control via enhanced NO bioavailability (Cosby et al., 2003; Ferguson et al., 2013; Sindler et al., 2014).

A brief single muscle contraction elicits a hyperemic response that results from rapid local vasodilation, and therefore serves as a tool to assess contraction‐induced hyperemia eliminating the influence of other stimulus for hyperemia (Clifford and Tschakovsky, 2008). Studies have demonstrated that NO plays a significant role for contraction‐induced hyperemia (Casey et al., 2013; Crecelius et al., 2013). Vascular function can also be assessed by monitoring the hyperemic response to an ischemia–reperfusion paradigm (i.e., reactive hyperemia) (Flammer et al., 2012). While these paradigms traditionally have been applied in conduit arteries using Doppler ultrasound or plethysmography, recently brief contractions (Meyer et al., 2004; Damon et al., 2007; Towse et al., 2011) and ischemia–reperfusion (Ledermann et al., 2006; Partovi et al., 2012,b) have also been used in combination with BOLD MR imaging to assess microvascular function in skeletal muscle.

The aim of this study was to examine if acute supplementation (46 h after eccentric contractions) with an AO cocktail (vitamin C, vitamin E and alpha‐lipoic acid) or nitrate‐rich, BR would restore microvascular reactivity (measured via BOLD MR imaging) 48 h after eccentric contractions, in young men and women. It was hypothesized that 48 h after eccentric contractions: (1) acute ingestion of AO will restore contraction‐induced hyperemia and reactive hyperemia via scavenging of ROS, and (2) acute ingestion of BR will restore contraction‐induced hyperemia and reactive hyperemia via increased NO availability.

## Methods

### Participants

Thirty healthy, young men (*n* = 18) and women (*n* = 12) volunteered to participate in the study. Because the study used within‐subject analysis, no adjustments were used for the phase of menstrual cycle for the female participants, as any influence of their menstrual cycle on microvascular function would be expected to be the same for the two experimental sessions (~48–72 h apart). A preliminary screening session was used to confirm that participants were (1) nonsmokers, (2) not taking any medications or supplements known to affect metabolism or blood flow, and (3) eligible for MR procedures (e.g., no metal implants). All participants were recreationally active, with a maximum of 3 h of structured exercise per week. Physical activity level was quantified by the short form of the International Physical Activity Questionnaire, and expressed as the Metabolic Equivalent Task minutes per week (MET‐min week^−1^). Experimental procedures and potential risks of the study were explained to all participants, who then provided informed consent, as approved by the Ethics Committee of North Denmark (N‐20130029) and in accordance with the Declaration of Helsinki.

### Experimental protocol

Participation in the study involved one habituation session, one exercise session involving eccentric muscle contractions, and two experimental sessions performed prior to and 48 h after the exercise session. Participants were instructed to refrain from strenuous physical activity, anti‐inflammatory medication, and antioxidant supplementation (e.g., vitamin pills) for 24 h prior to the first experimental session and throughout the duration of the study. Participants were randomized into three groups of 10 (4 women in each group) who, prior to the last experimental session ingested supplementation consisting of placebo (PL), AO or BR.

The two experimental sessions were performed at the MR Research Center at Aalborg University Hospital. To standardize conditions, these sessions were performed after an overnight fast, at the same time of day (±1 h) for each participant. The experimental procedures were performed in both legs and the order was identical between sessions. The order of experimental procedures was balanced, such that half of the participants started with CON, and the other half started with ECC.

### Habituation session

On the first visit (habituation), blood pressure was measured twice (Omron M4‐I, Omron Matsusaka, Japan), after a minimum of 15 min of rest, and the lowest reading was used for further analysis. Participants were then familiarized with the measure of pain pressure threshold (PPT), and the cuff occlusion procedure where a pressure cuff (VBM Medizintechnik GmbH, Sulz, Germany) was placed around the distal part of the thigh and inflated to 240 mmHg to prevent blood flow to the lower leg. Participants also practiced brief maximal voluntary contractions (MVCs) of the ankle dorsiflexor muscles to ensure that these contractions could be performed consistently.

### Eccentric exercise protocol

The exercise protocol consisted of controlled lengthening contractions of the dorsiflexor muscles of the experimental (ECC) leg, while the contralateral leg served as a nonexercising control (CON) leg (Gibson et al., 2006; Larsen et al., 2015). In brief, participants stood on a platform with the foot of the experimental leg attached to a footplate that was mounted to the platform, allowing free dorsiflexion and plantar flexion. The control leg was lifted off the platform, such that body weight was supported by the experimental leg. A controlled plantar flexion of the foot and ankle was performed until the footplate touched the floor. At this point, the control leg was extended and assisted in returning to the starting position. This routine was repeated 10 times per set, separated by 30 sec of rest, and continued until the participant was unable to perform controlled, lengthening contractions. To maintain balance during these contractions, the participant’s hands were placed flat on the wall (45 cm from the platform) at shoulder level. Half of the participants performed contractions with the right leg, while the other half used the left leg. Participants performed between 3 and 6 sets, as efficacy of this protocol varies across individuals. The protocol has previously shown to elicit moderate levels of muscle damage and muscle pain (Gibson et al., 2006; Larsen et al., 2015; Souza‐Silva et al., 2018).

### Supplementation

The supplementation consisted of 140 mL of beetroot juice (Beet it, James White Drinks, Ipswich, UK), an antioxidant cocktail consisting of capsules containing vitamin E, vitamin C, and alpha lipoic acid, or 140 mL of placebo drink. The beetroot juice contained 12.9 mmol of NO_3_^‐^ and was ingested 2 h prior to the first MR scan (effectively 2.5 and 3 h prior to MR scans in control leg and experimental leg). The dose and timing of BR supplementation was based on previous studies demonstrating peak levels of plasma NO_3_^‐^ 2–3 h post‐BR consumption (Wylie et al., 2013). To improve absorption, AO supplementation was ingested in two doses, 1.5 and 1 h prior to the first MR scan (Wray et al., 2012). The first dose consisted of 300 mg of alpha‐lipoic acid, 500 mg Vitamin C, and 200 I.U. Vitamin E, and the second dose consisted of 300 mg alpha‐lipoic acid, 500 mg Vitamin C, and 400 I.U. Vitamin E (Wray et al., 2012). The placebo drink consisted of 140 mL of water flavored with fruit juice and was consumed 2 h prior to the first MR scan. To prevent killing of anaerobic nitrate‐reducing bacteria on the dorsal surface of the tongue, participants were instructed not to brush their teeth or use mouth wash before or after ingesting the supplement.

### Magnetic resonance imaging

The MR imaging acquisitions were performed on a 3T MR scanner (Signa HDxt, General Electrics, Milwaukee, WI) using a 8‐channel extremity coil. Participants were positioned supine in the MR scanner. Padding was placed around the leg and knee to limit motion artifact and participant discomfort during the MR session. Participants rested in the scanner for approximately 15 min prior to the first MR scan in each session. Gradient echo images were acquired in 3 planes to locate the largest cross‐sectional area (CSA) of the TA muscle, and all subsequent images were acquired from this location. To ensure identical positioning between sessions, this location on the TA muscle was marked with a pen. Fast spin echo axial images [TR = 1500 msec, TE = 24 msec, echo train length = 4, FOV = 18 cm, slice thickness = 10 mm, number of slices = 3, slice gap = 1 mm, acquisition matrix 320 × 224, NEX = 1] were acquired to determine CSA by manually outlining the TA muscle. T2‐weighted images [TR = 2000 msec, TE = 20 msec, number of echoes = 4, FOV = 21 cm, slice thickness = 3.6 mm, number of slices = 3, slice gap = 5 mm, acquisition matrix 256 × 128, NEX = 1] were acquired to determine transverse relaxation time (T2), using T2 mapping within the outlined TA muscle. The MR image analyses were conducted by an experienced radiographer using standard software (AW server 2.0/AW VolumeShare 4, GE Healthcare, Milwaukee, WI).

### Hyperemic response to contraction and cuff occlusion using BOLD imaging

To assess contraction‐induced hyperemia, one‐shot gradient echo images [TR = 1000 msec, TE = 40 msec, FOV = 18 cm, slice thickness = 10 mm, acquisition matrix 64 × 64, NEX = 1, flip angle = 90°] were acquired continuously for 7.5 min while brief MVCs were performed every 60 sec (a total of 7 contractions) (Larsen et al., 2015). Verbal cues and encouragements were provided to ensure consistent timing and maximal effort of the contractions. Subsequently, a similar gradient echo protocol was used to monitor reactive hyperemia. Specifically, images were acquired continuously during 30 sec of rest, 5 min of cuff occlusion, and 2 min of recovery. The cuff positioned around the thigh was manually inflated to 240 mmHg within 3–4 sec and deflated within 1–2 sec.

**Figure 1:**
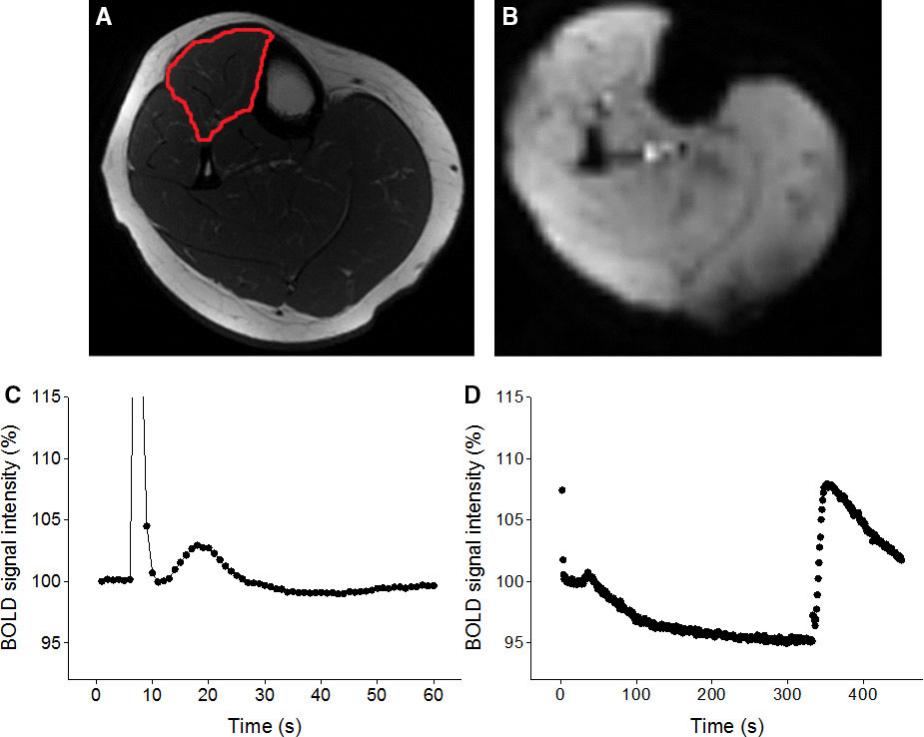
Representative examples of MR image (A) used to quantify muscle cross‐sectional area (outlined) and a BOLD MR image acquired (in the same slice) every second (B) to capture the hyperemic responses to brief maximal voluntary contraction (average of 7 contractions; C) and 5 min of cuff occlusion (D). Spike in signal intensity during contraction results from changes in signal saturation when the muscle contracts and moves in the imaged slice.

A custom‐written MatLab program was used to analyze the BOLD images (Larsen et al., 2015). In brief, a manually drawn region of interest (ROI) that comprised the majority of the TA muscle compartment and excluded bone, subcutaneous fat and resolved vessels was stored, and the mean signal intensity within the ROI was extracted for each of the 450 images. For the MVC protocol, the data were temporally aligned and averaged across all 7 contractions to create an individual 60‐sec time course for BOLD signal intensity (Fig. 1). The contraction artifact was omitted from the analysis. Peak change from minimum signal intensity postcontraction (%), time‐to‐peak (TTP, s), and time‐to‐half recovery (T_1/2_, s) of the BOLD transient were extracted from the time course. For the cuff protocol, including 450 images, peak change (relative to baseline; %), and TTP (from cuff deflation; s) were extracted (Fig. 1).

### Muscle strength and force recordings

With participants positioned supine in the MR scanner, the foot was attached to the foot plate (fixed at 120° ankle angle) of a custom‐built, MR compatible ergometer (Larsen et al., 2015). In brief, the footplate was connected to a force transducer (SSM‐AJ‐1000, Interface, Scottsdale, AZ) and the signal from the transducer was amplified, filtered, sampled at 100 Hz, and stored in a computer. To determine muscle strength, participants performed 3 MVCs of 3–5 sec durations, with 1 min of rest between each of these contractions. The force data acquired during the BOLD protocol (i.e., brief MVCs) were analyzed to determine peak force (% MVC), time–tension–integral (TTI, Ns), and contraction duration (s) using custom written MatLab program (The Mathworks, Natick, MA), as described previously (Larsen et al., 2015).

### Muscle pain

A modified Likert scale questionnaire (0: no soreness, 6: severe soreness) was used to evaluate pain in the TA muscle of ECC at baseline and every 24 h for 6 days after the eccentric exercise protocol. A handheld algometer (Somedic, Horby, Sweden) with a 1‐cm^2^ probe was oriented perpendicular to the belly of the TA muscle while pressure stimulation gradually increased (constant rate of 30 kPa sec^−^^1^). Participants were asked to press a button connected to the algometer when the pressure sensation became painful. The procedure was repeated twice, and the average was used for further analyses. The PPT was measured in both ECC and CON at baseline and 48 h after the eccentric exercise protocol. To eliminate possible influence of the pressure stimulation on muscle microvascular function, the PPT measures were obtained after the MR scans.

### Blood sample analyses

After completing each of the experimental sessions, and still in the fasted state, a 10 mL blood sample was obtained from the antecubital vein. Blood samples were immediately centrifuged, and plasma samples were placed in aliquots and stored at –80°C until analysis. Quantification of interleukin (IL) −10 and tumor necrosis factor (TNF)‐*α* were determined by using the MILLIPLEX Multiplex Map Human cytokine panel assay (Merck Millipore, Darmstadt, Germany) on a Luminex 200 platform (Luminex, TX). Data were analyzed with the Exponent software (Luminex, TX). Creatine kinase (CK) was assessed using an assay kit based on enzyme coupled reactions (Abnova, Taipei, Taiwan). To test the efficacy of the supplementations, total nitrite (R&D Systems, MN), and vitamin C (MyBioSource, CA) were assessed using ELISA kits. All analyses were performed in duplicate and the averages of these measurements were used for further analyses.

### Statistics

Statistical analyses were done using SPSS (IBM Corp., Armonk, NY). All data are presented as means and standard deviations (SD) with statistical significance established at *P* < 0.05. Normality of data was tested using Shapiro–Wilk tests. Two‐way (sex, group) ANOVAs were used to compare age, body mass index (BMI), blood pressure and physical activity level. Effects of the eccentric exercise protocol on mCSA, T2, muscle strength, PPT, and measures of microvascular function were analyzed separately for each leg (CON, ECC), using three‐way mixed ANOVAs with two between‐subjects factors of group (PL, AO, BR) and sex, and one within‐subjects factor of time (baseline, 48 h). Blood sample variables were analyzed using three‐way mixed ANOVAs with two between‐subjects factors of group (PL, AO, BR) and sex, and one within‐subjects factor of time (baseline, 48 h). Due to nonnormal distribution of blood sample variables, a log transformation was applied to the absolute values prior to analyses. Further effects were examined using Bonferroni‐adjusted post hoc tests. Likert scores of pain were analyzed using separate Friedman tests for each group. In case of significance, differences from baseline were identified with Wilcoxon signed‐rank tests using Bonferroni corrections. Differences in pain Likert scores across groups at 48 h were tested using a Kruskal–Wallis test. Pearson’s product‐moment correlation coefficients were used to explore significant associations.

## Results

All 30 participants completed the study. There were no effects by sex, group, or sex‐by‐group interactions for age, BMI, diastolic blood pressure or physical activity level. However, there was an effect by sex for systolic blood pressure (*P* < 0.004), such that the women had lower systolic blood pressure than the men. Participant characteristics are presented in Table 1.

**Table 1:** Group characteristics.

	PL		AO		BR	
	Men	Women	Men	Women	Men	Women
Age (years)	24.0 ± 2.0	24.0 ± 3.4	22.2 ± 2.7	22.5 ± 3.3	23.2 ± 2.0	22.8 ± 2.6
Body mass index (kg/m^2^)	24.1 ± 3.4	21.7 ± 2.3	22.9 ± 2.9	21.9 ± 2.3	23.2 ± 2.1	21.9 ± 4.2
Systolic BP (mmHg)*	126.0 ± 4.7	114.5 ± 8.0	121.5 ± 6.2	111.7 ± 4.7	125.5 ± 15.1	111.3 ± 9.4
Diastolic BP (mmHg)	70.0 ± 12.1	78.8 ± 8.2	71.2 ± 9.6	79.3 ± 1.5	73.5 ± 9.3	66.5 ± 4.1
Physical activity (MET‐min/wk)	2898 ± 1997	3183 ± 1929	2769 ± 1821	1915 ± 966	2693 ± 2403	3246 ± 1190

Data are presented as means ± SD. Main effect of sex (*P < 0.05). PL, placebo; AO, antioxidants; BR, beetroot juice; MET, metabolic equivalent task, BP, blood pressure.

### Muscle pain and muscle damage

**Figure 2:**
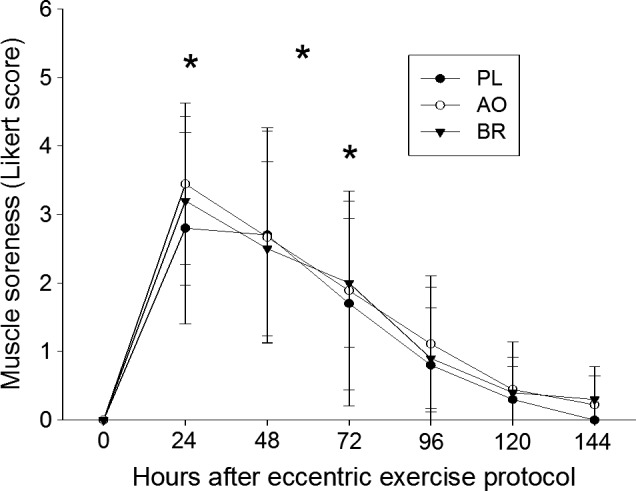
Likert scores, reflecting delayed onset muscle pain, in the tibialis anterior of the eccentrically exercised leg. Data are presented as means ± SD. Significant differences (*P < 0.05) from baseline (0 h) are indicated for placebo (PL), antioxidants (AO) or beetroot juice (BR) supplementation.

**Figure 3:**
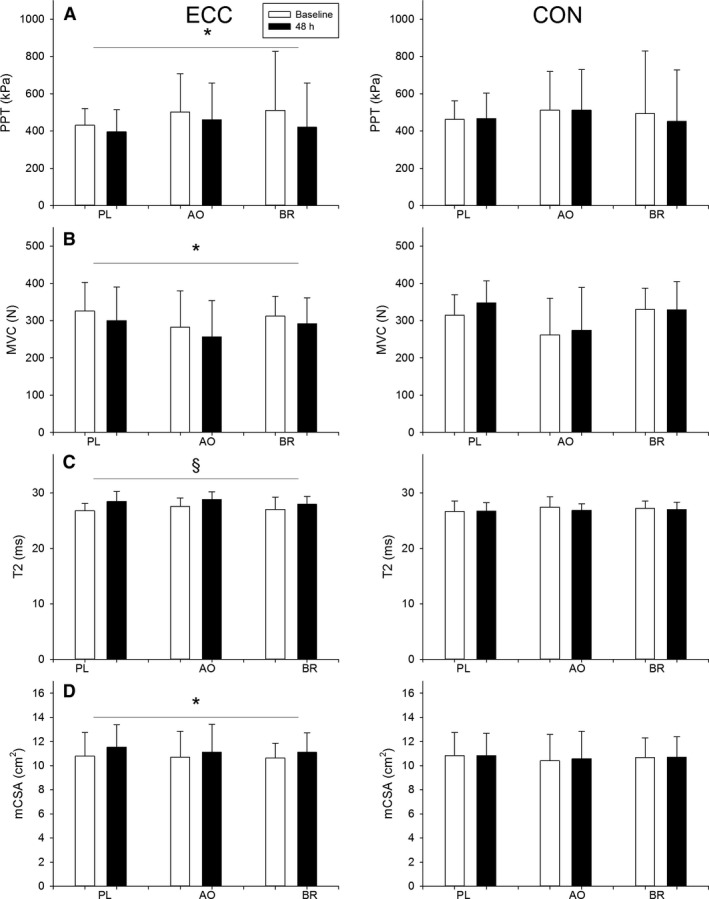
Markers of muscle damage obtained from the experimental leg (ECC) and the control leg (CON). Data for pain pressure threshold (PPT), muscle strength, transverse relaxation time (T2), and muscle cross‐sectional area (mCSA) are presented at baseline (open bars) and at 48 h (filled bars) after the eccentric exercise protocol. Data are presented as means ± SD. Effect of time (*P < 0.001), Sex‐by‐time interaction (§, effect of time in men only, *P* < 0.001).

All participants developed TA muscle pain of ECC, as indicated by Likert scores ≥ 1. Compared with baseline, higher Likert scores were found at 24, 48 and 72 h (*P* < 0.05) for all groups (Fig. 2). At 48 h, there were no differences in Likert scores across groups (*P* < 0.83). For PPT in ECC, there was an effect of time (*P* < 0.005) such that PPT was reduced at 48 h, with no other main effects or interactions (*P* > 0.16; Fig. 3A). For muscle strength in ECC, there was an effect of time (*P* < 0.001) such that strength was reduced at 48 h, and a main effect of sex (*P* < 0.01) such that the women were weaker than the men (Fig. 3B). There were no effects of group or interactions on strength in ECC (*P* > 0.49). For T2 in ECC, there was a time‐by‐sex interaction (*P* < 0.02; Fig. 3C). Post hoc tests revealed that T2 in ECC increased in the men (*P* < 0.001) but not in the women (*P* < 0.12). Also, T2 was higher in the women compared to the men at baseline (*P* < 0.002) but not at 48 h (*P* < 0.26). For mCSA in ECC, there was an effect by time (*P* < 0.001) such that mCSA was larger at 48 h, and an effect by sex (*P* < 0.001) such that the men had larger mCSA than the women (Fig. 3D). There were no effects by group or interactions for mCSA in ECC (*P* ≥ 0.06). In CON, there were main effects of sex on mCSA (*P* < 0.001), strength (*P* < 0.01), and T2 (*P* < 0.02) such that the women had smaller muscles, were weaker, and exhibited higher T2 compared with the men. There were no other main effects or interactions (*P* > 0.09).

### BOLD response to brief contraction

**Figure 4:**
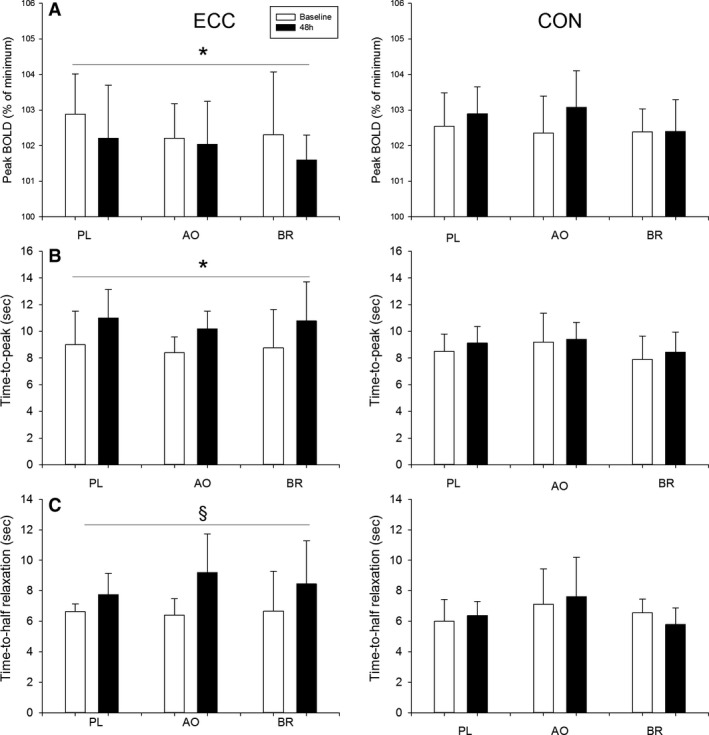
Peak, time‐to‐peak (TTP) and time‐to‐half relaxation (T_1/2_) of the BOLD response to brief maximal voluntary contraction are presented in the experimental leg (ECC) and control leg (CON). Data are presented at baseline (open bars) and 48 h (filled bars) after eccentric exercise protocol. Data are presented as means ± SD. Significant effect of time (*P < 0.001); Sex‐by‐time interaction (§, effect of time in women (*P* < 0.001) and in men (*P* = 0.02)).

Minimal BOLD responses to brief MVCs (<101% of minimum postcontraction) precluded analyses of hyperemic responses in two male participants in the PL group and one male participant in the BR group. Figure 1 shows a representative example of the BOLD responses to brief MVCs and cuff occlusion. Results from the BOLD responses to MVCs are presented in Figure 4. For peak BOLD in ECC, there was an effect of time (*P* < 0.04) such that peak BOLD was lower at 48 h, with no other main effects or interactions (*P* > 0.69, Fig. 4A). For TTP in ECC, there was an effect of time (*P* < 0.001) such that TTP was delayed at 48 h, with no other main effects or interactions (*P* > 0.83, Fig. 4B). For T_1/2_ in ECC, there was a time‐by‐sex interaction (*P* < 0.05, Fig. 4C). Post hoc analyses revealed that the prolongation of *T*_1/2_ at 48 h was evident in both the women (*P* < 0.001) and the men (*P* < 0.02). In CON, there were no main effects or interactions for peak, TTP or *T*_1/2_ of the BOLD response to MVC (*P* > 0.06).

### BOLD responses to cuff occlusion

**Figure 5:**
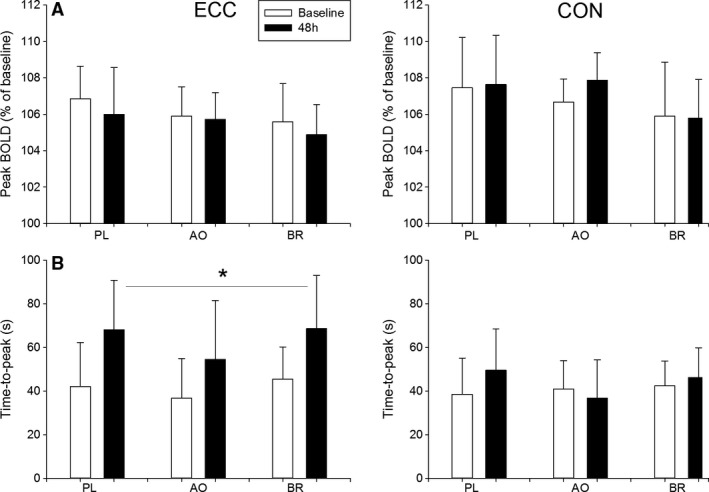
Peak, and time‐to‐peak (TTP) of the BOLD response, after cuff release, obtained in the experimental leg (ECC) and control leg (CON). Data are presented at baseline (open bars) and at 48 h (filled bars) after the eccentric exercise protocol. Data are presented as means ± SD. Significant effect of time (*P < 0.001).

Results from the BOLD response to cuff release are presented in Figure 5. For peak BOLD after cuff release in ECC, there were no main effects or interactions in ECC (*P* > 0.13, Fig. 5A). For TTP after cuff release in ECC, there was an effect of time (*P* < 0.001) such that TTP was prolonged at 48 h, and an effect of sex (*P* < 0.001) such that TTP was faster in the women compared with the men (Fig. 5B). There were no other main effects or interactions for TTP in ECC (*P* > 0.16). In CON, there was a group‐by‐sex interaction (*P* < 0.02) for peak BOLD. Post hoc analyses revealed that, in PL, peak BOLD after cuff release was greater in the women compared with the men (*P* < 0.03). Also, in the women, peak BOLD after cuff release was greater in PL compared with BR (*P* < 0.02). There were no other interactions or main effect for peak BOLD after cuff release in CON (*P* > 0.33). In CON, there was an effect of sex (*P* < 0.001) such that TTP after cuff release was shorter in the women compared with the men, with no other main effects or interactions (*P* > 0.10).

### Force during MVC protocol

Force data acquired during the brief contractions are presented in Table 2. For contraction level in ECC, there were no main effects or interactions (*P* > 0.14). For contraction duration in ECC, there were no main effects or interactions (*P* > 0.28). For TTI in ECC, there was an effect of time (*P* < 0.03) such that TTI was lower at 48 h, and a main effect of sex (*P* < 0.001) such that the women had lower TTI than the men. There were no other main effect or interactions (*P* > 0.13) for TTI in ECC. In CON, there was an effect of group on contraction level (*P* < 0.03), and post hoc analyses revealed that contraction level in CON was higher in AO compared with BR (*P* < 0.05). For contraction duration in CON, there were no main effects or interactions (*P* > 0.24). For TTI in CON, there was an effect of sex (*P* < 0.001) such that the women had lower TTI than the men, with no other main effects or interactions (*P* > 0.08).

**Table 2:** Force variables from brief maximal contractions.

	*ECC*	PL		AO		BR		*CON*	PL		AO		BR	
		Baseline	48 h	Baseline	48 h	Baseline	48 h		Baseline	48 h	Baseline	48 h	Baseline	48 h
MVC (%)		94.8 ± 5.4	99.4 ± 6.8	95.4 ± 8.7	93.8 ± 12.7	96.1 ± 7.5	93.6 ± 9.5	^†^	94.6 ± 3.7	98.7 ± 4.2	98.5 ± 13.1	96.0 ± 5.6	92.5 ± 6.0	89.3 ± 5.7
TTI (Ns)	*	430.2 ± 113.1	421.7 ± 95.0	385.6 ± 145.8	335.6 ± 150.6	383.9 ± 48.0	350.8 ± 95.6		421.8 ± 58.3	491.7 ± 99.9	356.0 ± 156.9	356.2 ± 157.7	393.1 ± 79.8	391.4 ± 80.5
Duration (s)		2.9 ± 0.1	3.0 ± 0.2	2.9 ± 0.2	2.9 ± 0.3	2.8 ± 0.2	2.9 ± 0.2		2.9 ± 0.1	2.9 ± 0.2	3.0 ± 0.3	2.9 ± 0.2	2.8 ± 0.3	2.9 ± 0.3

Data are presented as means ± SD. Main effect of time (*P < 0.05); main effect of group (^†^AO > BR, *P* < 0.05). PL, placebo; AO, antioxidants; BR, beetroot juice; MVC, maximal voluntary contraction; TTI, time–tension integral.

### Blood sample analyses

Results from blood samples analyses are presented in Table 3. For CK, there was an effect of sex (*P* < 0.001) such that CK was lower in the women compared to the men, with no other main effects or interactions (*P* > 0.21). For TNF*α*, there was an effect of time (*P* < 0.003) such that TNF*α* was lower at 48 h, with no other main effects or interactions (*P* > 0.06). For IL‐10 there were no main effects or interactions (*P* > 0.33). For total nitrite, there was a time‐by‐group interaction (*P* < 0.001). Post hoc analyses revealed that nitrite increased at 48 h in BR only (*P* < 0.001). Analysis of vitamin C was unsuccessful as >95% of the samples were below the detection threshold.

**Table 3:** Blood plasma levels.

	PL		AO		BR	
	Baseline	48 h	Baseline	48 h	Baseline	48 h
Creatine kinase (U/L)	159.6 ± 82.3	169.6 ± 133.9	194.3 ± 93.1	259.2 ± 220.9	161.1 ± 91.2	139.7 ± 69.1
IL‐10 (pg/mL)	15.1 ± 12.6	12.1 ± 9.2	14.7 ± 25.7	14.8 ± 25.8	6.6 ± 5.8	8.8 ± 5.9
TNF*α* (pg/mL)*	5.9 ± 3.6	3.9 ± 1.7	5.7 ± 3.6	4.7 ± 3.2	7.1 ± 4.5	5.8 ± 3.6
Total nitrite (*µ*mol/L)^†^	15.3 ± 9.5	17.1 ± 9.0	24.9 ± 38.1	18.8 ± 11.6	25.4 ± 19.5	309.0 ± 91.0^‡^

Data are presented as means ± SD. Significant effect of time (*P < 0.003); group‐by‐time interaction (^†^ *P* < 0.001); ^‡^Different from baseline (*P* < 0.001). PL, placebo; AO, antioxidants; BR, beetroot juice; TNF, tumor necrosis factor; IL, interleukin.

### Associations

At baseline, there were significant associations between the BOLD responses to brief MVC and postocclusion hyperemia. Specifically, there were modest associations for peak BOLD (*r* = 0.38, *P* < 0.005; *n* = 54) and TTP (*r* = 0.40; *P* < 0.003; *n* = 54), suggesting that there are factors that are independent of one another contributing to the two different hyperemic responses measured with BOLD MR imaging. Notably, there was an association between changes in peak BOLD responses to MVC and postocclusion reactive hyperemia (*r* = 0.47, *P* < 0.01; *n* = 27). Changes in peak reactive hyperemia were associated with changes in T2 (*r* = −0.45; *P* < 0.01; *n* = 30), with no other significant associations between changes in markers of muscle damage and changes in parameters from the BOLD responses (*P* > 0.07).

## Discussion

A novel aspect of this study was the use of acute supplementation with AO (Vitamin C, Vitamin E, alpha‐lipoic acid) or nitrate‐rich BR to test the hypotheses that oxidative stress and NO bioavailability are involved in impaired microvascular reactivity after eccentric exercise. Contrary to our hypotheses, AO and BR did not significantly recover the hyperemic responses to brief contraction or cuff occlusion. Furthermore, the hyperemic responses were maintained in the contralateral nonexercising leg, suggesting that impairment in the microvasculature was confined to the eccentrically exercised muscle tissue. Local impairment in the microvasculature after eccentric contractions may diminish the capability for prompt adjustments in muscle blood flow during activities that are intermittent or requiring rapid changes in oxygen delivery, which may have implications for overall muscle function.

### Impaired microvascular reactivity after eccentric contractions

Across all three groups (PL, AO, BR), eccentric contractions delayed TTP, reduced peak and prolonged *T*_1/2_ of the BOLD response to brief MVCs. These results confirm previous results (Larsen et al., 2015) and are consistent with reports of slowed hemodynamics at the onset of repeated muscle contractions (Kano et al., 2005), attenuated ADP‐stimulated vasodilation in arterioles (Heap et al., 2006), and augmented O_2_ delivery‐to‐O_2_ usage during rest‐to‐exercise transitions (Davies et al., 2008) following strenuous eccentric exercise. The significant reduction in peak BOLD at 48 h is in contrast to the results from a previous study (Larsen et al., 2015). The relative changes in peak BOLD were similar between studies, and the discrepancy may thus be explained by a larger sample size in this study (*n* = 30 vs. *n* = 14). To further investigate the effects of eccentric contractions on microvascular function, we assessed reactive hyperemia using BOLD MR imaging (Partovi et al., 2012). In agreement with the results from brief MVCs, TTP of the postocclusive BOLD response was markedly delayed (~54%) at 48 h. These results extend previous findings of attenuated FMD in conduit arteries following eccentric exercise (Stacy et al., 2013; Caldwell et al., 2016) by showing slowed reactive hyperemia in the microvasculature. Delayed TTP of the postocclusive BOLD response have been demonstrated in patients with peripheral arterial disease (Ledermann et al., 2006), systemic sclerosis (Partovi et al., 2012) and smokers (Nishii et al., 2015), compared to age‐matched controls, indicating that delayed TTP indeed is related to dysfunction of the microvasculature.

### Effects of beetroot juice and antioxidants

Hellsten *et al.* (Hellsten et al., 1997) reported that the endothelium of microvessels is the main site of ROS production and inflammation in response to eccentric exercise, suggesting that the microvasculature is prone to injury following eccentric exercise. To evaluate the specific role of oxidative stress and reduced NO bioavailability on impaired microvascular function after eccentric contractions, AO or BR supplementation was ingested 46 h after the eccentric contractions. A single dose of this particular AO cocktail has proven effective in reducing plasma‐free radicals and acutely restoring flow mediated dilation in individuals with elevated levels of oxidative stress (Richardson et al., 2007; Donato et al., 2010; Wray et al., 2012). Similarly, a single dose of beetroot juice has been demonstrated to acutely elevate plasma levels of nitrate and nitrite (Wylie et al., 2013), and acutely increase blood flow (via local vasodilation) during handgrip exercise (Richards et al., 2018). Plasma nitrate levels were elevated (~12‐fold) at 48 h with BR, verifying effective absorption and conversion of dietary nitrate and providing an alternative and abundant source of NO. However, data from this study do not verify a biological effect of dietary nitrate or presence of nitrate or antioxidants in the target tissue. The BOLD response was not recovered with BR, suggesting that impaired microvascular reactivity after eccentric contractions is independent of NO bioavailability. While no prior studies have examined the effects of BR, or other sources of NO_3_^‐^, on vascular function after eccentric exercise, Webb *et al.* (2008) showed that ingestion of a single dose of BR (23 mmol NO_3_^−^) prevented a decline in FMD in response to ischemia–reperfusion injury (20 min ischemia + 20 min reperfusion). Notably, in that study, BR was ingested prior to ischemia–reperfusion injury, and timing may thus influence the efficacy of BR in preserving vascular function in various models of tissue injury. Therefore, it is possible that ingestion of supplements prior to or immediately after the eccentric contractions could have prevented the impairment in microvascular function. Further, there is evidence to suggest that dietary nitrate elicits greatest effects on the vasculature supplying type II muscle fibers (Ferguson et al., 2013). Therefore, the findings from the dorsiflexor muscles (primarily composed of type I fibers), in this study, may not be generalized to all muscles.

Plasma levels of vitamin C could not be assessed in this study. The analyses were performed twice, but >95% of the samples were below the detection threshold for the assay. Possible explanations include lack of plasma acidification and long‐term storage (~2 years) in the freezer that may have resulted in substantial degradation of vitamin C (Karlsen et al., 2007). Hence, it is not possible to verify the efficacy of the AO supplementation. However, all participants confirmed that supplementation was ingested, as instructed. Several studies have demonstrated significant increases in vitamin C using similar timing of intake of this particular AO cocktail (Richardson et al., 2007; Donato et al., 2010; Wray et al., 2012). Furthermore, these studies have also documented the efficacy of this AO cocktail in scavenging ROS, also in young adults (Richardson et al., 2007; Donato et al., 2010; Wray et al., 2012). Hence, assuming that the AO cocktail indeed was effective, these results demonstrate no significant recovery of the BOLD responses in the AO group, suggesting that impaired microvascular reactivity after eccentric contractions is not restored with acute ingestion of AO.

### Possible mechanisms of impaired microvascular function

Kano and colleagues (Kano et al., 2004; Kano et al., 2005) reported collapsed capillaries and fewer capillaries supporting flowing red blood cells 48 h after strenuous eccentric contractions in rat muscle. Hence, structural changes within the microvasculature may result in fewer vessels available to accommodate an increase in blood flow, which provide a possible explanation for delayed and blunted BOLD response to brief contraction and cuff release in this study. Mechanical compression of vessels (as occur during brief contraction) elicits rapid vasodilation that may serve as feedforward mechanism to increase blood flow prior to effects of local vasodilating substances (Kirby et al., 2007). This rapid vasodilator response is likely due to intrinsic mechanosensitive mechanisms (i.e., myogenic response) within the endothelium or smooth muscle cells (Kirby et al., 2007). Therefore, a diminished myogenic response to a brief contraction may contribute to the delayed and blunted BOLD response in the present study.

### Markers of muscle damage

The timing of supplementation ingestion (~46 h postexercise) limited a potential influence of the specific supplementation on tissue degeneration and regeneration processes in response to eccentric muscle contractions, but allowed for examining the acute effect of supplementation on microvascular function. As intended, markers of muscle damage were not different across groups. The increase in mCSA of ECC manifests edema within the exercised muscle tissue 48 h after eccentric contractions. The swelling of the muscle tissue is likely accompanied by increased intramuscular pressure, which could restrict dilation of the microvasculature and thus contribute to delayed and blunted BOLD responses to brief contraction and cuff occlusion. Furthermore, increased T2 of TA in ECC provides compelling evidence of inflammation and muscle damage localized to the exercised muscle tissue. Specifically, ultrastructural changes of muscle and plasma levels of intracellular proteins (e.g., CK) have been reported to be strongly correlated with increases in muscle T2 after eccentric exercise (Nurenberg et al., 1992; Larsen et al., 2007). The interpretation of inflammation and muscle damage localized to the exercised TA is further supported by a reduction in strength and hyperalgesia (i.e., lower PPT). Taken together, these changes within the exercised tissue could possibly stimulate mechano‐ and metaboreceptor afferents and thus elicit adrenergic vasoconstriction through enhanced sympathetic outflow (Vissing, 1997). While no measure of muscle sympathetic nervous activity was included, the preserved BOLD response in the TA muscle of the contralateral leg, however, argues against increased sympathetic outflow as a mechanism for impaired microvascular reactivity, as sympathetic‐mediated vasoconstriction would have occurred in both legs (Vissing, 1997; Burton et al., 2016).

There was only a modest association between increases in T2 and decreases in peak BOLD reactive hyperemia, with no other significant correlations between markers of muscle damage and changes in parameters from the BOLD responses, suggesting a complex relationship between markers of muscle damage and changes in microvascular function. Future studies are warranted to verify if severity of muscle damage correlates with the degree of impairments in microvascular function. Importantly, the present results demonstrate that even moderate levels of muscle damage elicit significant impairments in microvascular function, which highlights the physiological and clinical relevance of the current findings.

The prolonged T_1/2_ of the BOLD response to brief MVCs reinforce that eccentric exercise alters the balance between O_2_ delivery and O_2_ usage, which is consistent with reports of increased muscle blood flow during submaximal exercise (Laaksonen et al., 2006) and augmented O_2_ delivery‐to‐O_2_ usage during rest‐to‐exercise transitions (Davies et al., 2008) after eccentric exercise. While diminished O_2_ diffusion capacity has been proposed as a mechanism (Davies et al., 2008), the marginal O_2_ deficit elicited by a single, brief contraction (Towse et al., 2011) questions the role of diffusion limitations for prolonged T_1/2_ in this study. Alternatively, inhibition of stretch‐activated cation channels has been shown to prolong the duration of reactive dilation in isolated arterioles (Koller and Bagi, 2002). Thus, altered function of mechanosensitive channels located in the vessel wall, possibly via structural changes, may provide a plausible explanation for altered regulation of vasomotor tone and prolonged T_1/2_ of the BOLD response to brief contraction.

### Experimental considerations

This study was strengthened by inclusion of measures in the TA of the contralateral, nonexercising leg, allowing for interpretation of the results in regard to both local and systemic effects. The eccentric contractions involved a relatively small muscle mass (i.e., TA in a single leg), which likely explains why plasma levels of CK and inflammatory cytokines were not elevated at 48 h (Sayers and Clarkson, 2003; Barnes et al., 2010). The preserved hyperemic responses in the contralateral leg show that impairment in microvascular function was confined to the exercised muscle tissue, which is consistent with the lack of a systemic inflammatory response. This interpretation is consistent with reports of reduced FMD (Caldwell et al., 2016) and lower peak blood flow (Souza‐Silva et al., 2018) isolated to arteries located in close proximity to the eccentrically exercised tissue.

The inclusion of both men and women, matched for age and physical activity level, allowed for examining possible sex differences. At baseline, the women exhibited faster TTP of BOLD reactive hyperemia, evident in both legs, indicating enhanced microvascular reactivity. This observation extends previous reports of greater brachial artery FMD (Levenson et al., 2001) and increased vasodilator responsiveness (Parker et al., 2007) in women. In contrast to the men, the women showed no increase in T2, indicating less muscle damage (Nurenberg et al., 1992; Larsen et al., 2007). There was also a tendency for a time‐by‐sex interaction (*P* = 0.06) for mCSA, suggesting less muscle edema in women compared to the men. However, other markers of muscle damage (i.e., PPT and strength) were not different between men and women, suggesting a similar response to eccentric contractions. Consistent with our results, others have also reported smaller changes in some, but not all, markers of muscle damage following eccentric exercise in women compared with men (Dannecker et al., 2012; Hicks et al., 2016). The changes in BOLD responses at 48 h were not different between men and women, suggesting that the effects of eccentric exercise on microvascular function do not vary by sex. The small sample sizes, however, presents a limitation of the study, and may have masked possible sex differences in the responses to eccentric contractions.

Lower force production (i.e., TTI) during the brief MVCs after eccentric contractions may have contributed to lower peak BOLD at 48 h (Meyer et al., 2004). Meyer and colleagues (Meyer et al., 2004) showed significant smaller peak BOLD response after brief isometric contractions of the dorsiflexor muscles performed at 50% compared with 100% MVC. However, monitoring the hyperemic responses to a series of brief isometric contractions of the dorsiflexor muscles (ranging from 10 to 100% MVC), Wigmore et al. (2004) reported a plateau in the postcontraction hyperemic response at ~60% MVC. Taken together, these results indicate that the modest decline in TTI of the brief MVCs at 48 h (~8%) did not influence the stimulus for contraction‐induced hyperemia and thus the interpretation of the BOLD response. Notably, lower force output during brief contractions would elicit faster TTP (Sanchez et al., 2009) and faster *T*_1/2_ (Carlson et al., 2008; Crecelius et al., 2013) of the hyperemic response, highlighting the physiological significance of delayed TTP and prolonged T_1/2_. The functional relevance of a 22–54% decline in microvascular reactivity is not clear, as various pathways may compensate for impaired microvascular reactivity and restore blood flow during continuous muscle activity. However, many activities comprise brief bursts of muscle activity that rely on the ability to rapidly adjust O_2_ delivery to match metabolic demand, emphasizing the physiological and clinical significance of these results.

## Conclusion

Impaired hyperemic responses to brief contraction and cuff occlusion in the microvasculature of the dorsiflexor muscles were found 48 h after eccentric contractions. Acute ingestion of antioxidants and nitrate‐rich, beetroot juice did not significantly restore the hyperemic responses in the presence of moderate level of muscle damage. These results demonstrate that acute administration of antioxidants or beetroot juice did not restore microvascular function after eccentric contractions, however timing of supplementation and choice of muscle group may have influenced the results. A possible explanation for impaired microvascular function after eccentric contractions involves structural changes within the microvasculature that render fewer vessels available to support blood flow. Future studies are warranted to examine if eccentric contractions elicit adaptations that protect the microvasculature from subsequent bouts of eccentric exercise.

## Conflict of Interest

The authors have no conflict of interest to declare.
